# Scaffold-Based Biomaterials for Periodontal Regeneration in Periodontitis: A Systematic Review and Meta-Analysis

**DOI:** 10.3390/jfb17060286

**Published:** 2026-06-08

**Authors:** Felicia Gabriela Beresescu, Simona Mucenic, Adriana Monea, Andrea Bors, Liana Beresescu

**Affiliations:** 1 Department of Teeth and Dental Arches Morphology, Faculty of Dental Medicine, George Emil Palade University of Medicine, Pharmacy, Science, and Technology of Targu Mures, 38 Gheorghe Marinescu Str., 540142 Targu Mures, Romania; 2 Department of Odontology and Oral Pathology, Faculty of Dental Medicine, George Emil Palade University of Medicine, Pharmacy, Science, and Technology of Targu Mures, 38 Gheorghe Marinescu Str., 540142 Targu Mures, Romania; 3 Department of Preventive and Community Dentistry, Faculty of Dental Medicine, George Emil Palade University of Medicine, Pharmacy, Science, and Technology of Targu Mures, 38 Gheorghe Marinescu Str., 540142 Targu Mures, Romania

**Keywords:** periodontal regeneration, scaffold-based therapy, tissue engineering, probing depth reduction, biomaterials

## Abstract

Background: Periodontitis is characterized by loss of the periodontal ligament, cementum, and alveolar bone. Scaffold-based biomaterials are intended to provide a three-dimensional framework for periodontal wound stabilization and tissue regeneration, but their incremental clinical benefit over conventional regenerative therapy remains uncertain. This systematic review and meta-analysis evaluated scaffold-based periodontal regenerative procedures for probing depth (PD) reduction, clinical attachment level (CAL) gain, and radiographic defect fill compared with conventional treatment. Methods: Original randomized controlled trials published from January 2020 to 1 March 2026 were searched in MEDLINE (Ovid), Embase, CENTRAL, and Web of Science, screened in Rayyan, and meta-analyzed in RevMan v5.4. Certainty was evaluated using GRADE. Results: Thirty-one studies were included. Scaffold-based interventions produced statistically significant but clinically modest PD reductions at 6 months (MD = −0.27 mm; 95% CI: −0.43 to −0.10; *p* = 0.001; I^2^ = 34%) and 12 months (MD = −0.21 mm; 95% CI: −0.41 to −0.01; *p* = 0.04; I^2^ = 22%), but not at 24 months. The overall PD effect was small (MD = −0.26 mm; *p* < 0.0001). CAL gain was not significant at 6 or 12 months but was significant at 24 months (MD = 1.00 mm; *p* < 0.0001; I^2^ = 0%). Defect fill improved at 12 months (MD = 0.51 mm; *p* = 0.02) but not at 6 months. Subgroup and meta-regression analyses did not identify significant effects of scaffold type or PRF/PRP enrichment (*p* > 0.05). Conclusions: Scaffold-based biomaterials may provide limited, time-dependent clinical and radiographic benefits as adjuncts to conventional periodontal regenerative therapy. The evidence remains constrained by heterogeneous interventions, modest effect sizes, low-to-very-low certainty for several outcomes, and a paucity of histologic confirmation of true periodontal regeneration.

## 1. Introduction

Periodontitis is a multifactorial, chronic inflammatory disease characterized by progressive destruction of tooth-supporting tissues, including alveolar bone, periodontal ligament (PDL), and cementum [1,2]. It is among the most prevalent oral diseases and a major cause of tooth loss in adults, with consequences for oral function, esthetics, and quality of life [1]. Although preventive measures and non-surgical treatment, including scaling and root planing with adjunctive antimicrobials when indicated, can control many cases, residual periodontal pockets may persist in advanced disease, particularly in Stage III–IV periodontitis [3].

Periodontal regeneration is the reformation of new cementum with inserted collagen fibers, functionally oriented periodontal ligament, and alveolar bone on a previously diseased root surface [4,5]. Conventional regenerative strategies, including guided tissue regeneration (GTR), bone grafting, and biologically active agents such as enamel matrix derivative (EMD), can improve probing depth (PD) and clinical attachment level (CAL), but outcomes vary with defect morphology, surgical protocol, and patient-related factors [6,7]. Predictable regeneration, therefore, remains challenging, especially in complex intrabony and furcation defects.

Periodontal tissue engineering aims to overcome limitations of conventional regeneration by combining biomaterial scaffolds, cells, and signaling molecules to support new attachment, bone formation, and functional periodontal restoration [8,9]. In this framework, a scaffold is not merely any adjunctive graft material; it is a three-dimensional, biocompatible construct that supports cell adhesion, migration, proliferation, differentiation, vascular ingrowth, and space maintenance during wound healing [10,11].

Emerging scaffold-based biomaterials are designed to approximate extracellular matrix architecture while controlling mechanical properties, bioactivity, and degradation [8,9]. Natural polymeric scaffolds, including collagen, chitosan, hyaluronic acid, and bacterial cellulose-based matrices, are generally biocompatible and bioactive but may exhibit limited mechanical stability or rapid degradation [8]. Synthetic polymers, including polylactic acid (PLA), polyglycolic acid (PGA), poly-lactic-co-glycolic acid (PLGA), and polycaprolactone (PCL), provide more tunable mechanical strength and degradation profiles, whereas composite scaffolds combine natural and synthetic phases to balance bioactivity with structural stability [12,13,14].

Recent advances in fabrication, particularly 3D printing and computer-aided design/computer-aided manufacturing (CAD/CAM) workflows, have enabled patient-specific scaffold designs that conform to the morphology of periodontal defects [15,16]. Such constructs may improve space maintenance and wound stability compared with conventional membranes and grafts. Hybrid, multiphasic, and compartmentalized scaffolds further reflect the biologic complexity of the periodontium by attempting to guide bone, PDL, and cementum regeneration in parallel [17].

Biofunctionalization of scaffolds with biologics is another active area of periodontal tissue engineering. Platelet-derived products such as platelet-rich plasma (PRP) and platelet-rich fibrin (PRF) provide autologous growth factors that may support angiogenesis and early wound healing [18,19]. Recombinant growth factors, including recombinant human platelet-derived growth factor (rhPDGF) and recombinant human bone morphogenetic protein-2 (rhBMP-2), have also been incorporated into scaffold or carrier systems to promote osteogenic and periodontal regenerative responses [20,21,22]. In this review, scaffold-based interventions were therefore distinguished from simple graft-plus-biologic protocols only when the material acted as an intended structural or space-maintaining framework.

Cell-based periodontal tissue engineering has also advanced, including approaches using mesenchymal stem cells (MSCs), periodontal ligament stem cells, conditioned medium, and other progenitor cell populations [23,24]. Although preclinical and early clinical findings are promising, translation remains limited by regulatory, logistical, manufacturing, and cost-related challenges [23,24].

Despite these advances, clinical evidence for scaffold-based periodontal tissue engineering remains fragmented. Studies differ substantially in scaffold composition, architecture, defect morphology, surgical technique, adjunctive biologics or cells, follow-up duration, and outcome assessment [9,25]. Moreover, most clinical trials rely on surrogate outcomes such as PD reduction, CAL gain, and radiographic defect fill, while few provide histologic confirmation of true regeneration, namely newly formed cementum, functionally oriented PDL, and alveolar bone.

Existing systematic reviews often focus on individual regenerative modalities, such as GTR membranes, biologic agents, or graft materials, rather than on scaffold-based biomaterials as an integrated tissue-engineering category [6,7]. A recent focus is justified because scaffold design, fabrication, biological enrichment, and imaging methods have changed rapidly since 2020, whereas earlier reviews do not consistently distinguish periodontal defects from peri-implant or ridge-preservation indications. This review, therefore, addresses a narrower clinical question: whether recent randomized evidence supports emerging scaffold-based biomaterials for the regeneration of tooth-supported intrabony and furcation periodontal defects.

The objective of this systematic review and meta-analysis was to evaluate the clinical, radiographic, and, when available, histologic effectiveness of recent scaffold-based biomaterials for periodontal tissue engineering in adults with periodontitis and tooth-supported intrabony or furcation defects, compared with conventional regenerative or surgical therapies.

## 2. Materials and Methods

The review was structured according to the Population–Intervention–Comparator–Outcome (PICO) framework, which is a systematic approach to planning and conducting research in health care.

PICO Framework

Population: Adults with chronic or aggressive periodontitis presenting intrabony (1–3-wall) or furcation defects, treated with regenerative surgery. Tooth-supported periodontium only; studies must report defect-level outcomes.

Intervention: Scaffold-based periodontal regenerative interventions, defined as natural, synthetic, or composite biomaterials intentionally used as a three-dimensional structural or space-maintaining framework to support periodontal tissue healing, with or without biologic enrichment (e.g., PRF/PRP, rhBMP-2, EMD, other growth factors, or bioactive molecules).

Comparison: Conventional regenerative or surgical controls, including open flap debridement, standard bone grafts or membranes, or scaling and root planing, when the control did not include an emerging scaffold construct as defined above.

Outcomes: Primary outcomes were probing depth (PD) reduction, clinical attachment level (CAL) gain, and radiographic or three-dimensional defect fill at 6, 12, and 24 months. Secondary outcomes were explored by scaffold type, biologic enrichment, surgical approach, and follow-up duration, where data permitted.

Study Design: randomized controlled trials.

Eligibility Criteria

Eligible studies were original randomized clinical trials in English, published between January 2020 and 1 March 2026, that evaluated scaffold-based biomaterials for periodontal regenerative surgery. To reduce conceptual ambiguity, a material was classified as a scaffold only when the trial described it as providing at least one structural tissue-engineering function: three-dimensional support, space maintenance, cell or clot stabilization, guided tissue ingrowth, controlled release, or defect-specific architecture. Bone substitutes, bone grafts, and barrier membranes were included only if they met this functional definition; interventions used solely as passive graft fillers or conventional membranes were excluded.

Exclusion criteria were non-original or non-full-text articles, non-English articles, in vitro, in silico, and ex vivo studies, non-randomized clinical designs, trials that did not measure periodontal regeneration outcomes, and trials without a scaffold-based biomaterial as defined above. Participants were adults with chronic or aggressive periodontitis or Stage III–IV periodontitis presenting intrabony and/or furcation defects in the tooth-supported periodontium; peri-implant sites and participants with systemic conditions likely to impair regeneration were excluded when reported.

Information Sources and Search Strategy

A customized electronic search was conducted in MEDLINE (via Ovid), Embase, the Cochrane Central Register of Controlled Trials (CENTRAL), and Web of Science (Clarivate) for records published from January 2020 to 1 March 2026. The search strategy combined free-text terms and controlled vocabulary (MeSH/Emtree/other subject headings) related to periodontitis, periodontal regeneration, and scaffold-based biomaterials, as detailed in Supplementary Table S1.

Study Selection

Following PRISMA guidelines (Supplementary Materials, Table S2), the inclusion and exclusion criteria were applied in two phases using Rayyan (https://www.rayyan.ai/). In the first phase, titles and abstracts were screened, and duplicates were identified and excluded within the platform. In the second phase, potentially eligible manuscripts underwent full-text screening. In both phases, two independent reviewers assessed each record and full-text article. Disagreements were resolved through discussion and, when necessary, consultation with a third reviewer. The protocol was registered to the INPLASY register under the identifier INPLASY202640058.

Data collection process

Eligible studies were extracted using a standardized electronic form created in Rayyan. The form captured the study population, defect type, scaffold characteristics, intervention and control procedures, outcome measures, and follow-up duration. Each report included was extracted by two reviewers. Differences were resolved by discussion and consensus; where consensus was not possible, a third reviewer adjudicated. When necessary, authors of included studies were contacted for clarification of unclear or missing information.

Data items

For each included study, outcome data were extracted on PD reduction, CAL gain, and radiographic/3D defect fill at 6, 12, and 24 months, where available. The latest reported time point within each major epoch was used. Defect- or site-level data were prioritized over tooth-level data; when studies mixed reporting levels or did not provide sufficient clustering information, no statistical adjustment for within-patient or within-tooth correlation was possible, and this limitation was recorded.

Participant characteristics, defect characteristics, scaffold composition, biologic enrichment, intervention and control procedures, sample size, follow-up duration, and funding source were also recorded for each study.

Risk of bias assessment

Risk of bias in each included randomized controlled trial was appraised independently by two reviewers using the Cochrane Risk of Bias 2 (RoB 2) tool for randomized trials [26]. The five mandatory domains evaluated were: (D1) bias arising from the randomization process; (D2) bias due to deviations from intended interventions; (D3) bias due to missing outcome data; (D4) bias in measurement of the outcome; and (D5) bias in selection of the reported result. Overall judgments followed the RoB 2 algorithm: High risk was assigned when at least one domain was rated high risk; Some concerns were assigned when at least one domain raised concerns; and Low risk was assigned only when all five domains were judged at low risk of bias.

Effect measures

For continuous outcomes, mean differences (MDs) with 95% confidence intervals were used as effect measures. When outcomes were reported as medians with interquartile ranges and could not be converted to means, they were summarized narratively rather than pooled.

Data synthesis

Studies were grouped for each synthesis according to the intervention and the reporting of comparable outcomes at similar follow-up intervals. Scaffold types were classified as natural, synthetic, or composite based on the principal material origin and composition; composite scaffolds included hybrid constructs combining natural and synthetic components or multi-phase biomaterials. This classification was operational and not intended to imply mechanistic equivalence, because scaffold architecture, carrier function, added biologics, defect morphology, and surgical protocol varied among trials. Priority was given to defect-level or site-level data, and studies lacking extractable defect- or site-level results were summarized narratively.

Individual study results were presented in tables summarizing participant and defect characteristics, scaffold type, biologic enrichment, surgical modality, and outcomes at each follow-up. Pooled effects and 95% confidence intervals for each outcome at 6, 12, and 24 months were presented as forest plots.

Random-effects meta-analysis models were used for quantitative synthesis because clinical and methodological heterogeneity was anticipated. Continuous outcomes were summarized as mean differences (MDs) with 95% confidence intervals. Statistical heterogeneity was assessed using the I^2^ statistic and Cochran Q test, with I^2^ values of 0–25%, 25–50%, and >50% interpreted as low, moderate, and substantial heterogeneity, respectively. RevMan v5.4 was used for meta-analysis. A two-sided *p* value < 0.05 was considered statistically significant.

Subgroup analyses were conducted by scaffold type and PRF/PRP enrichment to explore potential sources of heterogeneity. A subgroup analysis by surgical procedure and defect type was considered; however, sparse data within several surgical and defect categories, inconsistent reporting of defect morphology, and overlapping adjunctive strategies made a reliable pooled subgroup estimate inappropriate. These factors were therefore addressed narratively and in the limitations. Sensitivity analyses evaluated the robustness of pooled estimates by excluding studies at high risk of bias or with unclear allocation concealment or outcome assessment, by omitting one study at a time, and by restricting synthesis to studies with comparable follow-up windows.

Certainty assessment

Certainty in the body of evidence for each outcome was appraised according to GRADE principles, considering risk of bias, inconsistency, imprecision, and indirectness. Randomized trials initially provided high-certainty evidence, which was downgraded when substantial heterogeneity, wide confidence intervals, a small number of contributing studies, or important variation in defect type, surgical protocol, or follow-up period were present.

## 3. Results

**Figure 1:**
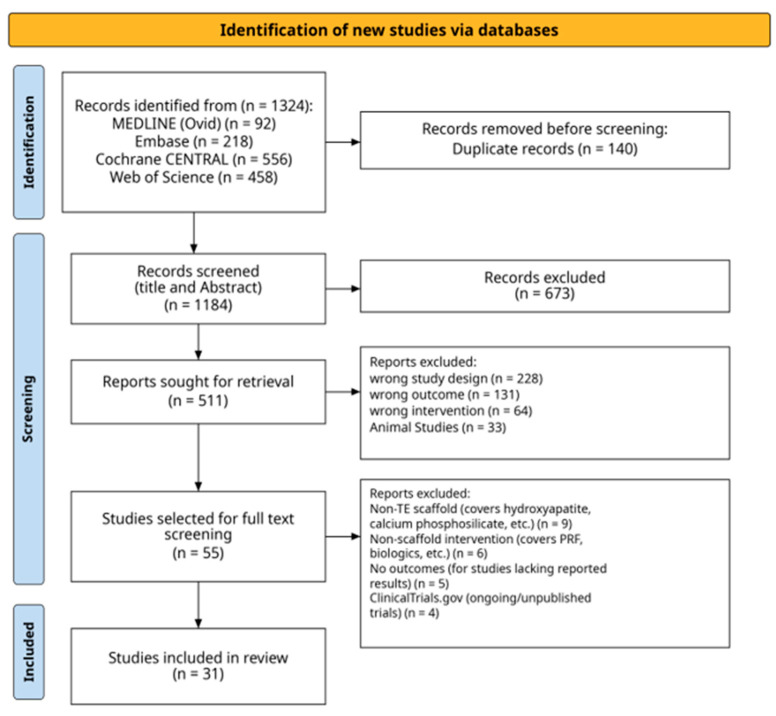
PRISMA flow diagram.

The literature search identified 1324 records from MEDLINE (Ovid; *n* = 92), Embase (*n* = 218), CENTRAL (*n* = 556), and Web of Science (WoS; *n* = 458). After duplicates were removed, 1184 records were screened by title and abstract; 673 were excluded and 511 reports were sought. Full-text assessment was completed for 55 studies, of which 24 were excluded for reasons including non-tissue-engineering scaffold materials (*n* = 9), non-scaffold-based interventions (*n* = 6), absence of relevant outcomes (*n* = 5), and ongoing or unavailable results (*n* = 4). Thirty-one studies [27,28,29,30,31,32,33,34,35,36,37,38,39,40,41,42,43,44,45,46,47,48,49,50,51,52,53,54,55,56,57] were included in qualitative synthesis and meta-analysis as shown in Figure 1.

Table 1 summarizes the characteristics of the 31 included studies on scaffold-based periodontal regeneration. Sample sizes ranged from 13 to 174 participants, totaling more than 1200 patients. Most studies involved patients with chronic periodontitis, while a smaller number included aggressive periodontitis or endo-perio lesions. Defects were predominantly intrabony, with morphologies ranging from 1 wall to 3 wall defects, including combined and furcation defects. The interventions included a wide variety of natural, synthetic, and composite scaffolds, and in some studies, biologically enriched constructs such as PRF/PRP, rhBMP-2, or dentin nanoparticles were evaluated. The predominant control groups were treated with conventional regenerative methodology, which comprised open-flap debridement, guided tissue regeneration with conventional membranes, and bone grafting procedures. The most frequently used surgical methods were guided tissue regeneration, open flap debridement, flap plus graft, and minimally invasive methods. The follow-up times ranged from 2 to 48 months, and most studies reported outcomes at 3, 6, 12, and 24 months.

Table 2 summarizes the scaffold composition, biologic enrichment, surgical technique, and outcome assessment methods across the included studies. Of the 31 studies, 15 used natural scaffolds, 10 used synthetic scaffolds, and 6 used composite scaffolds. Twelve studies evaluated scaffolds with PRF/PRP-based biologic enrichment, whereas the remaining studies used scaffolds without PRF/PRP, though several incorporated other adjuncts, such as enamel matrix derivative, rhBMP-2, fucoidan, melatonin, antibiotics, collagen, PLGA, RGD peptide, or herbal extract. Guided tissue regeneration was the most frequently used surgical technique, followed by open-flap debridement, flap-plus-graft procedures, minimally invasive surgical techniques, guided bone regeneration, standalone approaches, and scaling and root planing. Outcome assessment was primarily based on radiographic methods, including conventional radiographs, intraoral periapical radiographs, RVG, digital radiography, and CBCT, with several studies also incorporating clinical assessment.

**Figure 2:**
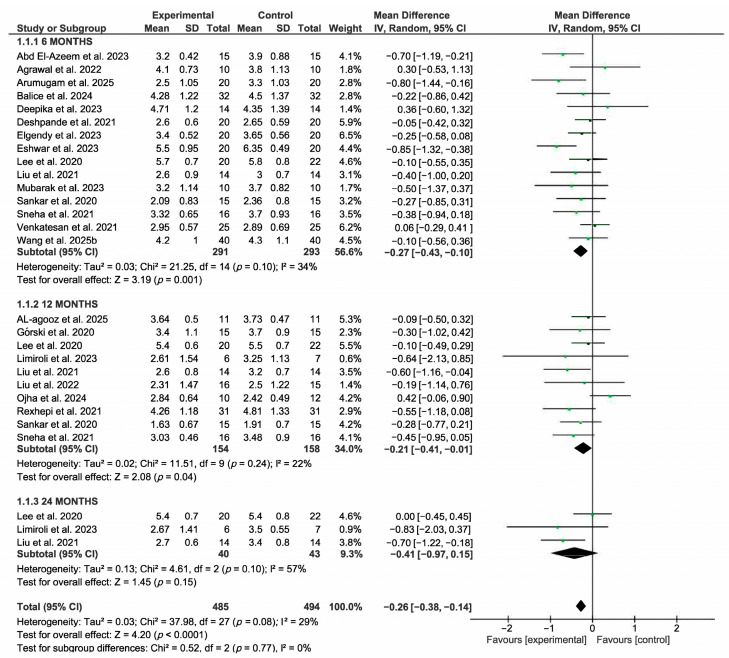
Forest plot of mean difference in probing depth reduction at 6, 12, and 24 months following scaffold-based periodontal regenerative interventions [27,29,31,33,34,37,39,42,43,44,45,46,47,48,49,50,52,53,54,55,57].

As shown in Figure 2, scaffold-based interventions produced statistically significant reductions in PD at 6 and 12 months compared with conventional therapy, although the absolute differences were clinically modest (<0.5 mm). The forest plot summarizes PD reduction at 6, 12, and 24 months. At 6 months, the pooled MD was −0.27 mm (95% CI: −0.43 to −0.10; Z = 3.19; *p* = 0.001), with low-to-moderate heterogeneity (I^2^ = 34%). At 12 months, the pooled effect remained significant (MD = −0.21 mm; 95% CI: −0.41 to −0.01; Z = 2.08; *p* = 0.04), with low heterogeneity (I^2^ = 22%). At 24 months, the pooled MD was −0.41 mm (95% CI: −0.97 to 0.15; Z = 1.45; *p* = 0.15), which was not statistically significant, with moderate heterogeneity (I^2^ = 57%). Across all time points, the combined effect was statistically significant but small (MD = −0.26 mm; 95% CI: −0.38 to −0.14; Z = 4.20; *p* < 0.0001).

**Figure 3:**
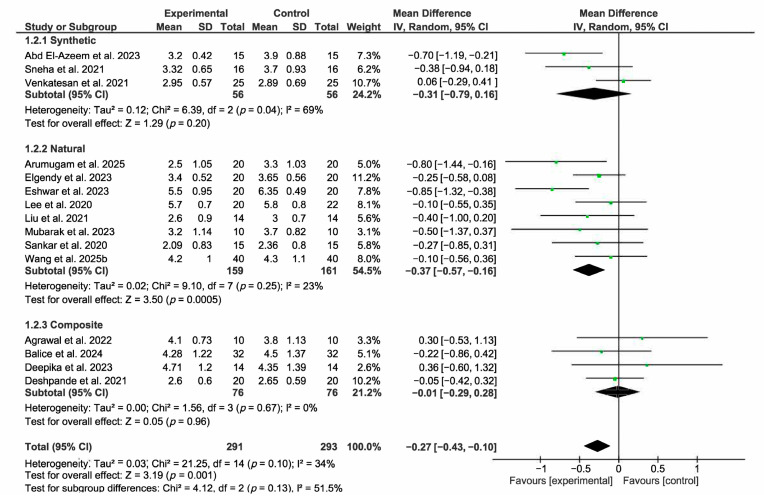
Subgroup analysis of probing depth reduction at 6 months by scaffold type in periodontal regenerative interventions [27,29,31,32,33,34,37,39,42,44,45,52,53,54,57].

Figure 3 shows the subgroup analysis of PD reduction at 6 months by scaffold type. Synthetic scaffolds showed a non-significant pooled MD of −0.31 mm (95% CI: −0.79 to 0.16; Z = 1.29; *p* = 0.20), with substantial heterogeneity (I^2^ = 69%). Natural scaffolds showed a statistically significant but small PD reduction (MD = −0.37 mm; 95% CI: −0.57 to −0.16; Z = 3.50; *p* = 0.0005), with low heterogeneity (I^2^ = 23%). Composite scaffolds showed a minimal, non-significant effect (MD = −0.01 mm; 95% CI: −0.29 to 0.28; Z = 0.05; *p* = 0.96; I^2^ = 0%). The test for subgroup differences was not significant (Chi^2^ = 4.12; df = 2; *p* = 0.13; I^2^ = 51.5%), suggesting that scaffold type did not reliably modify the effect at 6 months.

**Figure 4:**
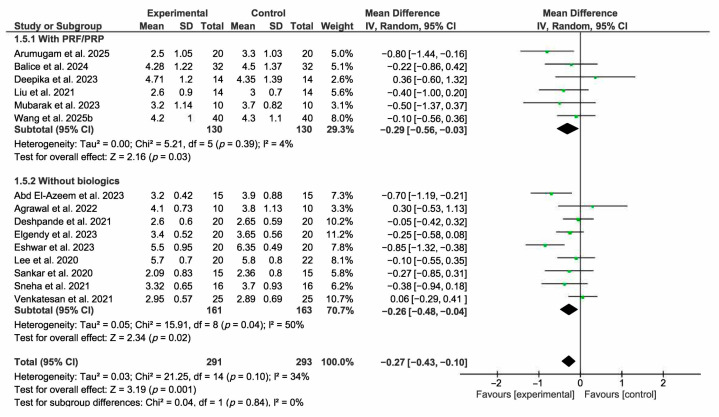
Subgroup analysis of probing depth reduction at 6 months by biologic enrichment (with vs. without PRF/PRP) in scaffold-based periodontal regenerative interventions [27,29,31,32,33,34,37,39,42,44,45,52,53,54,57].

Figure 4 presents the subgroup analysis of PD reduction at 6 months according to PRF/PRP enrichment. In sites treated with PRF/PRP, the pooled MD was −0.29 mm (95% CI: −0.56 to −0.03; Z = 2.16; *p* = 0.03), with minimal heterogeneity (I^2^ = 4%). For sites without PRF/PRP, the pooled PD reduction was −0.26 mm (95% CI: −0.48 to −0.04; Z = 2.34; *p* = 0.02), with moderate heterogeneity (I^2^ = 50%). The overall pooled PD reduction across both subgroups was −0.27 mm (95% CI: −0.43 to −0.10; Z = 3.19; *p* = 0.001; I^2^ = 34%). The test for subgroup differences was not statistically significant (Chi^2^ = 0.04; df = 1; *p* = 0.84; I^2^ = 0%), indicating that PRF/PRP enrichment did not significantly modify the effect of scaffold-based interventions on PD reduction at 6 months.

**Figure 5:**
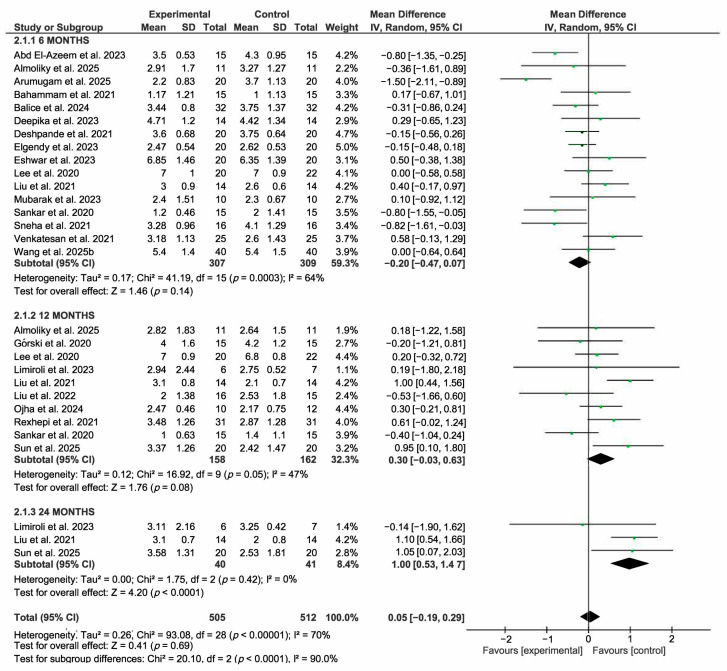
Forest plot of clinical attachment level (CAL) gain at 6, 12, and 24 months following scaffold-based periodontal regenerative interventions [27,29,30,31,32,33,36,37,39,41,42,43,44,45,48,49,50,52,53,54,57].

Figure 5 presents the pooled analysis of CAL gain at 6, 12, and 24 months. At 6 months, the pooled MD was −0.20 mm (95% CI: −0.47 to 0.07; Z = 1.46; *p* = 0.14), indicating no significant improvement in CAL compared with controls and substantial heterogeneity (I^2^ = 64%). At 12 months, the pooled effect was 0.30 mm (95% CI: −0.03 to 0.63; Z = 1.76; *p* = 0.08), also non-significant, with moderate heterogeneity (I^2^ = 47%). At 24 months, the pooled MD was 1.00 mm (95% CI: 0.53 to 1.47; Z = 4.20; *p* < 0.0001), showing statistically significant CAL gain with no heterogeneity (I^2^ = 0%). Overall, combining all time points, CAL gain was minimal and not significant (MD = 0.05 mm; 95% CI: −0.19 to 0.29; Z = 0.41; *p* = 0.69), with substantial heterogeneity (I^2^ = 70%). The significant test for subgroup differences by follow-up duration (Chi^2^ = 20.10; df = 2; *p* < 0.0001; I^2^ = 90%) indicates that timing strongly influenced observed CAL effects.

**Figure 6:**
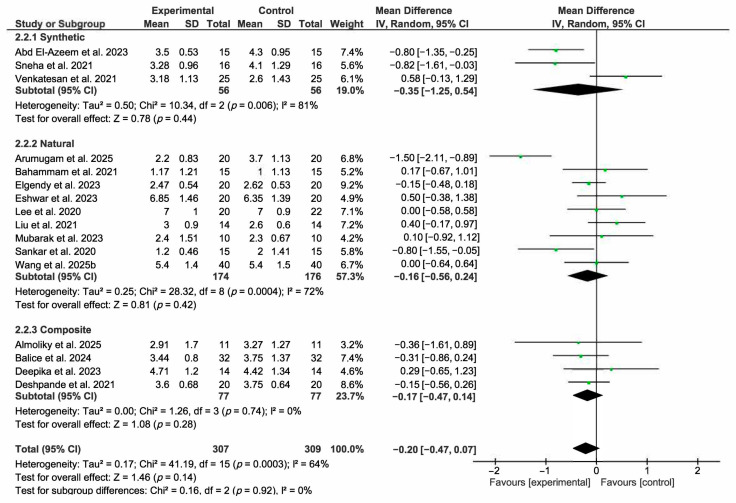
Subgroup analysis of clinical attachment level gain at 6 months by scaffold type in scaffold-based periodontal regenerative interventions [27,29,31,33,36,37,39,41,42,44,45,46,52,53,54,55].

Figure 6 presents the subgroup analysis of CAL gain at 6 months according to scaffold type. Synthetic scaffolds showed a non-significant pooled MD of −0.35 mm (95% CI: −1.25 to 0.54; Z = 0.78; *p* = 0.44), with substantial heterogeneity (I^2^ = 81%). Natural scaffolds had a pooled effect of −0.16 mm (95% CI: −0.56 to 0.24; Z = 0.81; *p* = 0.42), also non-significant, with substantial heterogeneity (I^2^ = 72%). Composite scaffolds showed a minimal, non-significant effect (MD = −0.17 mm; 95% CI: −0.47 to 0.14; Z = 1.08; *p* = 0.28; I^2^ = 0%). The test for subgroup differences was not significant (Chi^2^ = 0.16; df = 2; *p* = 0.92; I^2^ = 0%), suggesting that scaffold type did not significantly modify CAL gain at 6 months.

**Figure 7:**
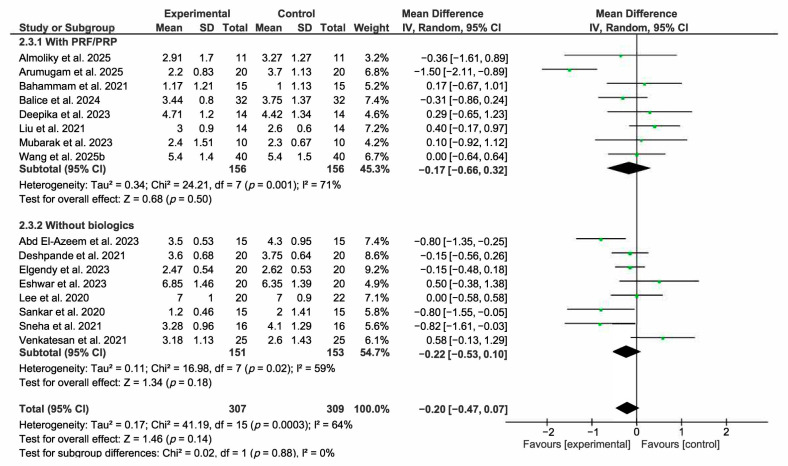
Subgroup analysis of clinical attachment level gain at 6 months by biologic enrichment (with vs. without PRF/PRP) in scaffold-based periodontal regenerative interventions [27,29,31,33,36,37,39,41,42,44,45,46,52,53,54,55].

Figure 7 presents the subgroup analysis of CAL gain at 6 months according to PRF/PRP enrichment. In sites treated with PRF/PRP, the pooled MD was −0.17 mm (95% CI: −0.66 to 0.32; Z = 0.68; *p* = 0.50), with substantial heterogeneity (I^2^ = 71%), indicating no statistically significant improvement compared with controls. For sites without PRF/PRP, the pooled CAL gain was −0.22 mm (95% CI: −0.53 to 0.10; Z = 1.34; *p* = 0.18), with moderate heterogeneity (I^2^ = 59%), also not statistically significant. The test for subgroup differences was not significant (Chi^2^ = 0.02; df = 1; *p* = 0.88; I^2^ = 0%), suggesting that PRF/PRP did not modify the effect of scaffold-based interventions on CAL gain at 6 months.

**Figure 8:**
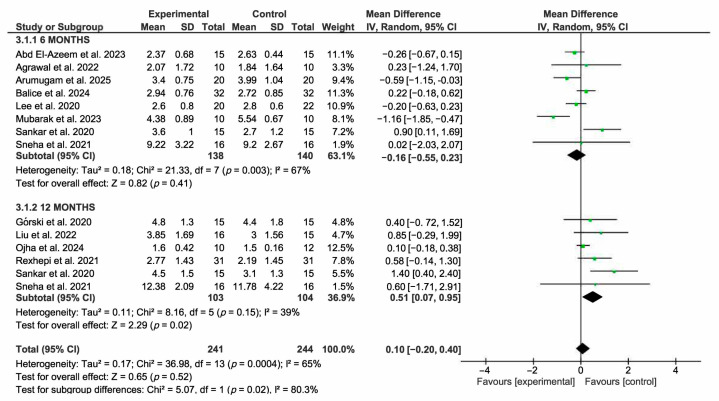
Forest plot of defect fills at 6, and 12 months following scaffold-based periodontal regenerative interventions [31,33,34,37,39,43,44,49,50,52,54,57].

Figure 8 presents the pooled analysis of defect fill at 6 and 12 months. At 6 months, the pooled MD was −0.16 mm (95% CI: −0.55 to 0.23; Z = 0.82; *p* = 0.41), with substantial heterogeneity (I^2^ = 67%), indicating no significant improvement compared with controls. At 12 months, the pooled MD was 0.51 mm (95% CI: 0.07 to 0.95; Z = 2.29; *p* = 0.02), with moderate heterogeneity (I^2^ = 39%), showing a statistically significant improvement. Across both time points, the combined MD was 0.10 mm (95% CI: −0.20 to 0.40; Z = 0.65; *p* = 0.52), with substantial heterogeneity (I^2^ = 65%). The test for subgroup differences was significant (Chi^2^ = 5.07; df = 1; *p* = 0.02; I^2^ = 80.3%), suggesting that follow-up duration influenced defect-fill outcomes. Sensitivity analysis at 6 months, excluding high-risk or methodologically uncertain studies, did not materially change the direction of the effect.

**Figure 9:**
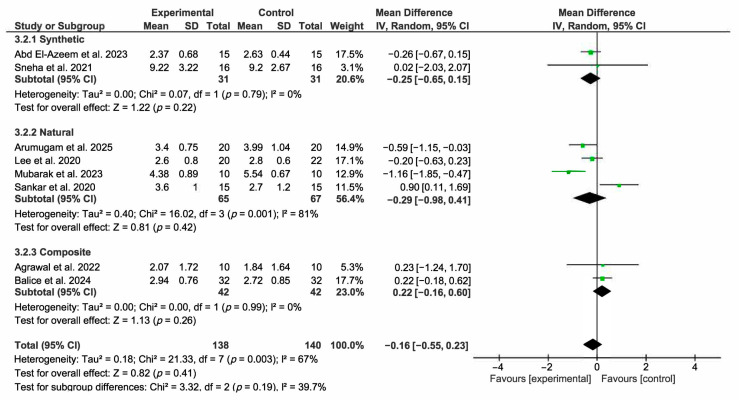
Subgroup analysis of defect fills at 6 months by scaffold type in scaffold-based periodontal regenerative interventions [31,33,34,37,39,44,52,54].

Figure 9 presents the subgroup analysis of defect fill at 6 months according to scaffold type. For synthetic scaffolds, the pooled mean difference was −0.25 mm (95% CI: −0.65 to 0.15; Z = 1.22; *p* = 0.22), with no heterogeneity (I^2^ = 0%), indicating a non-significant improvement compared with controls. Natural scaffolds had a pooled effect of −0.29 mm (95% CI: −0.98 to 0.41; Z = 0.81; *p* = 0.42), with high heterogeneity (I^2^ = 81%), and this effect was not statistically significant. Composite scaffolds showed a pooled effect of 0.22 mm (95% CI: −0.16 to 0.60; Z = 1.13; *p* = 0.26), with no heterogeneity (I^2^ = 0%), and was not significant. Overall, the pooled defect fill across all scaffold types was −0.16 mm (95% CI: −0.55 to 0.23; Z = 0.82; *p* = 0.41), with substantial heterogeneity (I^2^ = 67%). The test for subgroup differences was not significant (Chi^2^ = 3.32; df = 2; *p* = 0.19; I^2^ = 39.7%), suggesting that scaffold type did not significantly modify defect fill outcomes at 6 months.

**Figure 10:**
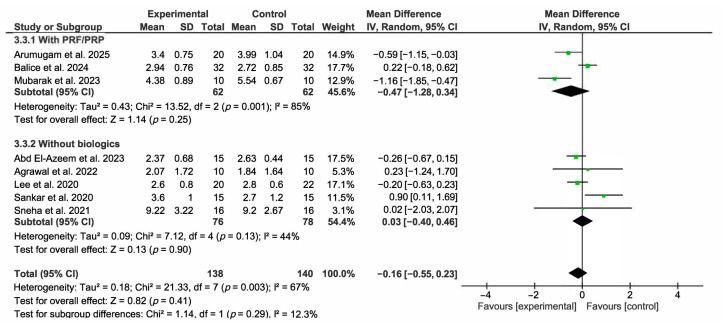
Subgroup analysis of defect fills at 6 months by biologic enrichment (with vs. without PRF/PRP) in scaffold-based periodontal regenerative interventions [31,33,34,37,39,44,52,54].

Figure 10 presents the subgroup analysis of defect fill at 6 months according to PRF/PRP enrichment. In sites treated with PRF/PRP, the pooled MD was −0.47 mm (95% CI: −1.28 to 0.34; Z = 1.14; *p* = 0.25), with substantial heterogeneity (I^2^ = 85%). For sites without PRF/PRP, the pooled effect was 0.03 mm (95% CI: −0.40 to 0.46; Z = 0.13; *p* = 0.90), with moderate heterogeneity (I^2^ = 44%). The test for subgroup differences was not significant, suggesting that PRF/PRP enrichment did not reliably modify defect fill at 6 months.

Table 3 presents the results of meta-regression analyses exploring the influence of scaffold type and PRF/PRP enrichment on 6-month outcomes. For PD reduction, none of the moderators was statistically significant: natural scaffold (MD = −0.26 mm; 95% CI: −0.72 to 0.21; *p* = 0.28), synthetic scaffold (MD = −0.25 mm; 95% CI: −0.72 to 0.23; *p* = 0.32), and PRF/PRP enrichment (MD = −0.16 mm; 95% CI: −0.58 to 0.25; *p* = 0.45). Similarly, scaffold type and PRF/PRP enrichment did not significantly predict CAL gain or defect fill. These analyses should be interpreted cautiously because the moderator categories were broad and did not account for scaffold architecture, carrier function, defect morphology, or surgical protocol.

Overall distribution of risk of bias

**Figure 11:**
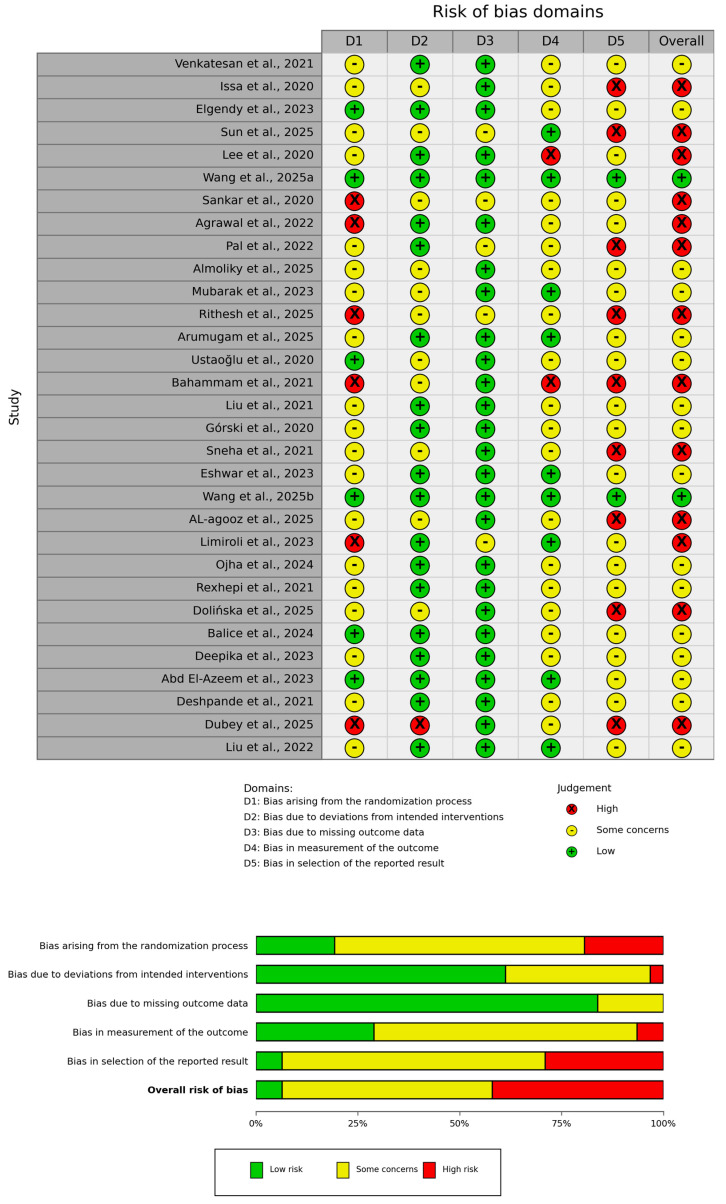
Risk of Bias Assessment of Included Randomized Controlled Trials (RoB 2) [27,28,29,30,31,32,33,34,35,36,37,38,39,40,41,42,43,44,45,46,47,48,49,50,51,52,53,54,55,56,57].

Figure 11 presents the overall risk-of-bias assessment for the 31 included trials. Three studies (9.7%) were classified as low risk overall (Wang et al., 2025a; Wang et al., 2025b; Abd El-Azeem et al., 2023) [32,46,54]. Most trials (*n* = 21, 67.7%) were judged to raise some concerns, largely attributable to incomplete reporting of allocation concealment and the inherent impossibility of operator blinding in open-label surgical interventions. Seven trials (22.6%) were classified as high risk of bias overall (Sankar et al., 2020; Rithesh et al., 2025; Bahammam et al., 2021; Sneha et al., 2021; Al-agooz et al., 2025; Dolińska et al., 2025; Dubey et al., 2025) [33,38,41,44,47,51,56], predominantly owing to concerns in the domains of randomization, outcome measurement, and selective reporting. None of the trials were classified as unclear in the general judgment.

The sensitivity analyses reveal that the high-risk, methodologically uncertain studies had the greatest effect, overstating statistical heterogeneity across syntheses without a significant substantive effect on the pooled effect estimates. The strength of the main findings of these studies is reassuring regarding the absence of outcome-level reporting bias as a significant risk to the validity of the meta-analytic results. However, the high level of heterogeneity, which remains despite their exclusion, highlights the true clinical and methodological heterogeneity of the trials included and is a cause to consider the interpretation of the pooled estimates.

Certainty of evidence

Table 4 summarizes the pooled outcome estimates and the certainty of the evidence for each outcome at 6, 12, and 24 months. The reduction in probing depth was statistically significant at 6 and 12 months, with moderate evidence certainty, and was non-significant at 24 months, with very low evidence certainty. The gain in clinical attachment level was not significant at 6 and 12 months, and at 24 months, the gain was statistically significant, but certainty was very low. In radiographic defect fill, there was no significant effect at 6 months and very low certainty, and a statistically significant effect at 12 months with low certainty.

## 4. Discussion

This systematic review and meta-analysis evaluated recent randomized controlled trials on scaffold-based biomaterials for periodontal tissue engineering in periodontitis. The findings indicate small, time-dependent benefits: PD reduction was statistically significant at 6 and 12 months, CAL gain was significant only at 24 months, and defect fill was significant only at 12 months. These effects support a potential adjunctive role for scaffold-based materials but do not demonstrate consistent superiority over conventional regenerative therapy.

Subgroup and meta-regression analyses did not show that broad scaffold category (natural, synthetic, or composite) or PRF/PRP enrichment significantly modified clinical outcomes. These null findings should not be interpreted as proof that scaffold design is irrelevant; rather, they reflect the limited ability of the available trials to isolate architecture, degradation profile, bioactive loading, defect morphology, and surgical protocol as separate determinants of regeneration.

The pooled PD reduction of approximately 0.2–0.3 mm is statistically significant but clinically modest. Recent systematic reviews of periodontal regenerative therapy likewise report that regenerative approaches can improve PD and CAL in intrabony defects, but the magnitude and durability of benefit vary according to defect characteristics, surgical technique, and patient-level factors [6,7]. Therefore, the observed PD benefit should be framed as an adjunctive effect rather than a clinically transformative improvement.

The short-term PD reduction may be explained by scaffolds’ structural function as temporary extracellular-matrix-like frameworks that stabilize the clot, preserve space, and facilitate cell migration and angiogenesis [8,9,10,11]. However, the clinical effect likely depends not only on material composition but also on porosity, pore interconnectivity, degradation kinetics, mechanical stability, bioactive loading, and surgical handling [20,58].

The absence of significant PD improvement at 24 months suggests that early benefits may not be sustained in all settings. Longer-term periodontal stability is strongly influenced by supportive periodontal therapy, plaque control, host response, smoking, systemic health, and maintenance adherence, in addition to the regenerative material itself [6,59,60].

CAL gain was non-significant at 6 and 12 months but significant at 24 months, suggesting a delayed clinical attachment response. True periodontal regeneration involves cementogenesis, functionally oriented PDL formation, and alveolar bone regeneration; these processes require more time than early pocket-depth reduction and may not be captured fully by short-term endpoints [4,5,23].

The delayed CAL signal should nevertheless be interpreted cautiously. The 24-month finding was based on fewer contributing studies than shorter follow-ups, and the overall CAL effect across all time points was not statistically significant. Thus, the data suggest a possible longer biological window for attachment recovery but do not prove that scaffold-based therapy consistently increases the magnitude of true periodontal regeneration [61,62].

Clinical heterogeneity was a central limitation. The included trials pooled intrabony defects, furcation defects, endo-perio lesions, chronic or aggressive periodontitis, and Stage III–IV periodontitis, as well as multiple surgical techniques and follow-up periods. Such diversity can dilute or exaggerate pooled estimates when trials are combined without adequate stratification by defect morphology, surgical protocol, baseline defect depth, and patient risk profile [6,25,63,64,65].

Defect fill was non-significant at 6 months but significant at 12 months, a pattern consistent with the slower kinetics of bone formation, matrix mineralization, and graft or scaffold consolidation. Nevertheless, radiographic defect fill should not be equated with true periodontal regeneration, because radiopaque repair tissue or bone fill does not necessarily demonstrate newly formed cementum with inserted PDL fibers [4,66,67].

Modern radiographic methods, particularly CBCT, can improve evaluation of defect topography and bone-fill percentage compared with conventional periapical imaging, but they remain indirect measures of tissue quality. Differences in image acquisition, calibration, measurement thresholds, and two-dimensional versus three-dimensional analysis may also contribute to between-study variability [68,69,70].

Accordingly, a conceptual distinction is essential: PD reduction and CAL gain indicate clinical improvement, and radiographic fill indicates hard-tissue repair or mineralized tissue gain, but none of these surrogate endpoints alone confirm complete periodontal regeneration. Histologic evidence remains the reference standard for demonstrating new cementum, functionally inserted PDL, and alveolar bone on previously diseased root surfaces [4,5].

Another key finding is that the broad scaffold category did not predict outcomes. Natural scaffolds showed favorable trends in some analyses, but these were not consistent or statistically robust. This challenges the assumption that natural, synthetic, or composite origin alone determines clinical success; design parameters and the context of use appear more important than material class alone [8,10,14,25].

Biologic enrichment with PRF/PRP also did not significantly modify pooled outcomes. PRF and PRP may release growth factors, such as PDGF, TGF-beta, and VEGF, that support early wound healing, but their long-term regenerative effects appear inconsistent across trials [19,71]. Similarly, other adjuncts such as EMD, rhBMP-2, melatonin, RGD peptide, antibiotics, and herbal extracts may have biologic rationale, but the current evidence base is too heterogeneous to isolate their independent effects [21,22,63].

The biomaterials represented in the review can be summarized as follows: natural scaffolds (e.g., collagen membranes, chitosan or fucoidan hydrogels, PRF-related matrices, dentin-derived materials, and autogenous or xenogenic matrices) emphasize biocompatibility and bioactivity; synthetic scaffolds (e.g., PLA, PLGA, PCL, calcium phosphate, and nanofiber constructs) emphasize tunable mechanics and degradation; and composite scaffolds attempt to combine biologic signaling with dimensional stability. This taxonomy is useful for reporting but insufficient for mechanisms, because two materials within the same category may differ substantially in porosity, stiffness, degradation, surface chemistry, and release kinetics [72,73].

Limitations

First, clinical heterogeneity was substantial. Studies differed in scaffold materials, architecture, defect types, surgical methods, adjunctive biologics, outcome measurement, and follow-up duration. This limits direct comparability and reduces the precision and biological interpretability of pooled estimates.

Second, many studies enrolled small samples, limiting statistical power and increasing the risk of imprecision and type II error. Some trials also had incomplete reporting of randomization, allocation concealment, blinding, or outcome assessment, which contributed to concerns about risk of bias.

Third, outcome reporting was inconsistent. Several studies did not report all primary outcomes, and some mixed tooth-, site-, and defect-level data were presented without sufficient information for statistical adjustment. Differences in examiner calibration and measurement methods may have contributed to additional variability.

Fourth, most studies were short- to medium-term, and few provided follow-ups beyond 24 months; the durability of any scaffold-related benefit therefore remains uncertain.

Fifth, patient-related modifiers such as smoking, oral hygiene, diabetes or other systemic conditions, defect anatomy, plaque control, and maintenance compliance were not consistently controlled or reported, although these factors can strongly influence periodontal healing.

The methodological limitations of this review should also be emphasized. Despite a comprehensive search strategy, publication bias could not be ruled out, and funnel-plot asymmetry could not be reliably assessed in several subgroups due to the small number of studies. The heterogeneity of interventions and outcomes prevented meta-analyses that were comparable across all endpoints, and subgroup analyses were limited by sparse data within broad categories. Most importantly, the evidence relied primarily on clinical and radiographic surrogate endpoints, whereas histologic confirmation of true periodontal regeneration was rare. Therefore, the findings support cautious clinical use and further research rather than definitive claims of complete regeneration.

Clinical implications

Clinically, scaffold-based biomaterials should be viewed as adjuncts to, rather than replacements for, established periodontal regenerative principles.

They may be most useful in selected cases, including:

- Intrabony defects with morphology that support space maintenance
- Complex defects requiring clot stabilization or compartmentalized healing
- Cases in which enhanced wound stability or soft-tissue healing are clinically desirable

However, clinicians should recognize that the observed incremental benefit is modest and long-term superiority over conventional therapy is not established. Treatment success depends heavily on defect morphology, surgical technique, plaque control, and patient compliance.

Therefore, scaffold selection should be individualized according to defect characteristics, patient risk profile, operator experience, availability, and cost-effectiveness rather than an expectation of uniformly superior outcomes.

Policy implications

The current evidence does not support routine adoption of any specific scaffold material as standard care for periodontal regeneration.

Policy and guideline development should emphasize:

- Standardized clinical protocols and outcome definitions
- Evidence-based selection of biomaterials with transparent indications
- Cost–benefit and feasibility considerations, particularly in low-resource settings

Regulatory bodies and funders should encourage high-quality clinical trials that evaluate emerging biomaterials before widespread implementation.

Future research directions

Future studies should address several evidence gaps:

- Large, adequately powered randomized controlled trials with standardized defect definitions, surgical protocols, and outcome measurements are needed to improve comparability.
- Long-term follow-up should be incorporated to determine whether early improvements are sustained.

Next-generation biomaterials should be investigated in clinically meaningful strata, including smart scaffolds with controlled drug or growth-factor delivery and patient-specific 3D-printed or CAD/CAM-designed scaffolds.

Bioactive nanomaterials and multiphasic constructs tailored to bone-PDL-cementum regeneration. Greater emphasis should be placed on patient-reported outcomes, quality of life, functional improvement, and cost-effectiveness. Histologic or validated three-dimensional evidence should be pursued where ethical and feasible to distinguish true regeneration from repair.

## 5. Conclusions

This review and meta-analysis indicate that scaffold-based biomaterials can provide adjunctive, time-dependent benefits in periodontal regeneration, but the magnitude and consistency of these effects remain limited. Across 31 recent randomized controlled trials, scaffold-based interventions produced statistically significant but modest PD reductions at 6 and 12 months, whereas CAL gain became significant only at 24 months, and radiographic defect fill was significant at 12 months. These findings suggest that scaffolds may contribute to improved wound stability, space maintenance, and local tissue support; however, they should be viewed as supportive regenerative tools rather than replacements for well-executed conventional periodontal surgery.

The evidence did not demonstrate a consistent clinical advantage for a particular scaffold category or for PRF/PRP enrichment. This result should be interpreted cautiously because the included trials differed in defect morphology, surgical protocols, comparator treatments, scaffold composition, biologic adjuncts, outcome definitions, follow-up duration, and risk of bias. Moreover, most studies relied on clinical and radiographic surrogates; the scarcity of histologic or validated three-dimensional evidence prevents firm conclusions about true regeneration of cementum, periodontal ligament, and alveolar bone rather than repair or defect fill alone.

For clinical practice, scaffold-based biomaterials may be considered on a case-by-case basis as adjuncts in carefully selected periodontal defects, with treatment planning guided by defect anatomy, patient-related risk factors, operator expertise, material availability, and cost-effectiveness. Future research should prioritize adequately powered, multicenter randomized trials with standardized scaffold descriptions, defect-specific stratification, calibrated outcome measures, longer follow-up, patient-reported outcomes, and histologic or validated three-dimensional endpoints where feasible. Such studies are necessary to determine which scaffold architectures, degradation profiles, bioactive components, and delivery strategies translate into clinically meaningful and durable periodontal regeneration.

## Figures and Tables

**Table 1:** Characteristics of Included Studies.

Author’s/Year	*N*	Periodontitis Type	Defect Type	Defect Morphology	Intervention	Control	Technique	Follow-Up (Months)
Venkatesan et al., 2021 [27]	50	Chronic	Intrabony	1–2 wall, combined	Amniotic membrane + BiCP	CM + BiCP	GTR	6
Issa et al., 2020 [28]	40	Chronic	Intrabony	2–3 wall	SMV gel + occlusive membrane	SMV + OM (modified)	OFD + GTR	6, 9
Elgendy et al., 2023 [29]	40	Stage III	Intrabony	2–3 wall	Autogenous dentin nanoparticles	Allograft (Maxgraft)	OFD	6
Sun et al., 2025 [30]	40	Chronic/ Aggressive	Intrabony	2–3 wall	CEBDX + M-MIST	M-MIST alone	M-MIST	12, 24
Lee et al., 2020 [31]	42	Not specified	Intrabony	1-wall	DPBM + EMD	DPBM alone	OFD	6, 12, 24
Wang et al., 2025a [32]	174	Stage III	Intrabony	1–3 wall	Crosslinked CM + DBBM	Non-crosslinked CM + DBBM	GTR	3, 6
Sankar et al., 2020 [33]	15	Furcation	Furcation	2–3 wall	Silk fibroin membrane + xenograft	CM + xenograft	Flap + graft	3, 6, 12
Agrawal et al., 2022 [34]	20	Chronic	Intrabony	2–3 wall	DFDBA + amniotic membrane	DFDBA + CM	GTR	3, 6
Pal et al., 2022 [35]	40	Stage II/III	Intrabony	2–3 wall	BCP + EMD	BCP alone	GTR	12
Almoliky et al., 2025 [36]	22	Stage III	Intrabony	1–2 wall	PRF + DFDBA	CM + DFDBA	GTR	3, 6, 9, 12
Mubarak et al., 2023 [37]	30	Stage III	Intrabony	2–3 wall	L-PRF + CM	OFD alone	GTR	6
Rithesh et al., 2025 [38]	24	Chronic/ Aggressive	Intrabony	3-wall	i-PRF + nano-HA graft	OFD + graft	OFD	3, 6
Arumugam et al., 2025 [39]	40	Chronic	Intrabony	2–3 wall	PRF plug	OFD + BCP	GTR	3, 6
Ustaoğlu et al., 2020 [40]	45	Endo-perio	Intrabony	2–3 wall	T-PRF + OFD	OFD alone	OFD	9
Bahammam et al., 2021 [41]	60	Chronic	Intrabony	2–3 wall	Not clearly defined scaffold	OFD alone	OFD	6
Liu et al., 2021 [42]	28	Aggressive	Intrabony	2–3 wall	BPBM + PRF	BPBM alone	GTR	6, 12, 24
Górski et al., 2020 [43]	30	Aggressive	Intrabony	1–3 wall	Perforated CM + xenograft	Standard CM + graft	GTR	12, 48
Sneha et al., 2021 [44]	32	Furcation	Furcation	2–3 wall	rhBMP-2 + collagen scaffold	PRF	GTR	6, 12
Eshwar et al., 2023 [45]	40	Chronic	Intrabony	2–3 wall	Fucoidan–chitosan hydrogel	CGF	Standalone	3, 6, 9
Wang et al., 2025b [46]	80	Chronic/ Aggressive	Intrabony	1–3 wall	RCCM + DBBM	NCM + DBBM	GTR	3, 6
Al-agooz et al., 2025 [47]	67	Chronic	Intrabony	3-wall	Melatonin nanoparticles	Placebo + SRP	SRP	6
Limiroli et al., 2023 [48]	13	Chronic	Furcation	2–3 wall	PLA membrane + Bio-Oss	EMD + Bio-Oss	GBR	12, 24
Ojha et al., 2024 [49]	22	Chronic	Intrabony	2–3 wall	CPC–PLGA composite	CPC cement	GTR	12
Rexhepi et al.,2021 [50]	62	Chronic/ Aggressive	Intrabony	1–2 wall	L-PRF + IBB	CM + IBB	Standalone	12
Dolińska et al., 2025 [51]	41	Stage III	Intrabony	1–3 wall	DBBM + autogenous bone	DBBM alone	GTR	6
Balice et al., 2024 [52]	64	Stage III–IV	Intrabony	1–3 wall	ABG + L-PRF	ABG + CM	Flap + graft	12
Deepika et al., 2023 [53]	28	Chronic	Intrabony	2–3 wall	HA + PRF + PLA/PGA	HA + membrane	GTR	6
Abd El-Azeem et al., 2023 [54]	45	Stage III–IV	Intrabony	2–3 wall	RGD hydrogel + MIST	MIST alone	MIST	6
Deshpande et al., 2021 [55]	40	Chronic	Intrabony	2–3 wall	nHAC	nHA	OFD	3, 6
Dubey et al., 2025 [56]	31	Chronic	Intrabony	2–3 wall	PCL nanofiber scaffold	Blank scaffold	SRP	2
Liu et al., 2022 [57]	31	Chronic	Intrabony	1–3 wall	DBBM + CM + MIST	MIST alone	MIST	12

**Table 2:** Scaffold composition, biologic enrichment, surgical technique, and outcome assessment methods.

Author’s/Year	Scaffold Type	Biologics/Enrichment	Biologic Enrichment	Surgical Technique	Outcome Assessment Method
Venkatesan et al., 2021 [27]	Synthetic	Without biologics	None	GTR	Intraoral radiographs
Issa et al., 2020 [28]	Synthetic	Without biologics	None	OFD + GTR	Radiographs
Elgendy et al., 2023 [29]	Natural	Without biologics	None	OFD	Digital radiographs
Sun et al., 2025 [30]	Natural	Without biologics	Not specified	M-MIST	CBCT, periapical radiograph
Lee et al., 2020 [31]	Natural	Without biologics	EMD	OFD	Not reported
Wang et al., 2025a [32]	Natural	Without biologics	Not specified	GTR	Periapical radiographs
Sankar et al., 2020 [33]	Natural	Without biologics	None	Flap + graft	Radiographs
Agrawal et al., 2022 [34]	Composite	Without biologics	None	GTR	IOPA radiographs
Pal et al., 2022 [35]	Synthetic	Without biologics	EMD	GTR	Radiographs
Almoliky et al., 2025 [36]	Composite	With PRF/PRP	PRF	GTR	Radiographs, clinical
Mubarak et al., 2023 [37]	Natural	With PRF/PRP	PRF	GTR	Radiographs, CBCT
Rithesh et al., 2025 [38]	Synthetic	With PRF/PRP	PRF	OFD	RVG radiographs
Arumugam et al., 2025 [39]	Natural	With PRF/PRP	PRF	GTR	Radiographs, CBCT
Ustaoğlu et al., 2020 [40]	Natural	With PRF/PRP	PRF	OFD	Two-dimensional radiograph
Bahammam et al., 2021 [41]	Natural	With PRF/PRP	PRF	OFD	Radiographs
Liu et al., 2021 [42]	Natural	With PRF/PRP	PRF	GTR	Radiographs, CBCT
Górski et al., 2020 [43]	Natural	With PRF/PRP	PRF	GTR	Radiographs, CBCT
Sneha et al., 2021 [44]	Synthetic	Without biologics	rhBMP-2	GTR	Digital radiography
Eshwar et al., 2023 [45]	Natural	Without biologics	Fucoidan	Standalone	Radiographs, CBCT
Wang et al., 2025b [46]	Natural	With PRF/PRP	PRF	GTR	Radiographs, CBCT
Al-agooz et al., 2025 [47]	Synthetic	Without biologics	Melatonin	SRP	CBCT, clinical
Limiroli et al., 2023 [48]	Synthetic	Without biologics	EMD	GBR	CBCT
Ojha et al., 2024 [49]	Synthetic	Without biologics	PLGA	GTR	Radiographs, CBCT
Rexhepi et al., 2021 [50]	Natural	With PRF/PRP	PRF	Standalone	Radiographs, CBCT
Dolińska et al., 2025 [51]	Natural	Without biologics	Antibiotics	GTR	Radiographs, CBCT
Balice et al., 2024 [52]	Composite	With PRF/PRP	PRF	Flap + graft	Radiographs, CBCT
Deepika et al., 2023 [53]	Composite	With PRF/PRP	PRF	GTR	CBCT
Abd El-Azeem et al., 2023 [54]	Synthetic	Without biologics	RGD peptide	MIST	CBCT, clinical
Deshpande et al., 2021 [55]	Composite	Without biologics	Collagen	OFD	Radiographs, CBCT
Dubey et al., 2025 [56]	Synthetic	Without biologics	Herbal extract	SRP	Clinical, CBCT
Liu et al., 2022 [57]	Composite	Without biologics	None	MIST	Clinical, radiographic

**Table 3:** Meta-regression of scaffold type and biologic enrichment on 6-month probing depth reduction, clinical attachment gain, and defect fill in periodontal regeneration.

Outcome	Moderator	Estimate (MD)	SE	z-Value	*p*-Value	95% CI
PD Reduction	Intercept	0.002	0.178	0.009	0.993	−0.348, 0.351
	Natural Scaffold	−0.256	0.239	−1.072	0.284	−0.723, 0.212
	Synthetic Scaffold	−0.246	0.244	−1.004	0.315	−0.724, 0.233
	Biologics (PRF/PRP)	−0.161	0.212	−0.760	0.448	−0.576, 0.254
CAL Gain	Intercept	−0.254	0.329	−0.770	0.441	−0.899, 0.392
	Natural Scaffold	0.203	0.404	0.502	0.616	−0.589, 0.995
	Synthetic Scaffold	−0.092	0.466	−0.198	0.844	−1.005, 0.821
	Biologics (PRF/PRP)	−0.044	0.381	−0.116	0.907	−0.791, 0.703
Defect Fill	Intercept	0.753	0.556	1.354	0.176	−0.337, 1.842
	Natural Scaffold	−0.702	0.532	−1.319	0.187	−1.744, 0.341
	Synthetic Scaffold	−0.851	0.691	−1.233	0.218	−2.206, 0.503
	Biologics (PRF/PRP)	−0.703	0.455	−1.544	0.123	−1.595, 0.190

**Table 4:** Summary of effect estimates and certainty of evidence (GRADE) by outcome and time point.

Outcome	Timepoint	Pooled MD (mm)	95% CI	*p*-Value	I^2^	Certainty (GRADE)
Probing Depth (PD) Reduction						
Probing Depth (PD) Reduction	6 months	−0.27 mm	−0.43 to −0.10	*p* = 0.001	34%	⊕⊕⊕⊝ MODERATE
Probing Depth (PD) Reduction	12 months	−0.21 mm	−0.41 to −0.01	*p* = 0.04	22%	⊕⊕⊕⊝ MODERATE
Probing Depth (PD) Reduction	24 months	−0.41 mm	−0.97 to 0.15	*p* = 0.15	57%	⊕⊝⊝⊝ VERY LOW
Clinical Attachment Level (CAL) Gain						
Clinical Attachment Level (CAL) Gain	6 months	−0.20 mm	−0.47 to 0.07	*p* = 0.14	64%	⊕⊝⊝⊝ VERY LOW
Clinical Attachment Level (CAL) Gain	12 months	0.30 mm	−0.03 to 0.63	*p* = 0.08	47%	⊕⊝⊝⊝ VERY LOW
Clinical Attachment Level (CAL) Gain	24 months	1.00 mm	0.53 to 1.47	*p* < 0.0001	0%	⊕⊕⊝⊝ LOW
Radiographic Defect Fill						
Radiographic Defect Fill	6 months	−0.16 mm	−0.55 to 0.23	*p* = 0.41	67%	⊕⊝⊝⊝ VERY LOW
Radiographic Defect Fill	12 months	0.51 mm	0.07 to 0.95	*p* = 0.02	39%	⊕⊕⊝⊝ LOW

## Data Availability

No new data were created or analyzed in this study. Data sharing is not applicable to this article.

## Supplementary Materials

The following supporting information can be downloaded at: https://www.mdpi.com/article/10.3390/jfb17060286/s1, Table S1: Literature Search Syntax. Table S2. PRISMA 2020 Checklist.

## Abbreviations

The following abbreviations are used in this manuscript:

rhBMP-2	Bone morphogenetic proteins
CAL	Clinical attachment level
CAD/CAM	Computer-aided design/computer-aided manufacturing
EMD	Enamel matrix derivative
GTR	Guided tissue regeneration
MSCs	Mesenchymal stem cells
PRF	Platelet-rich fibrin
PRP	Platelet-rich plasma
PLGA	Poly-lactic-co-glycolic acid
PCL	Polycaprolactone
PLA	Polylactic acid
PGA	Polyglycolic acid
PDL	Periodontal ligament
PD	Probing depth
rhPDGF	Recombinant human platelet-derived growth factor
3D	Three-dimensional
ECM	Tissue extracellular matrix
WoS	Web of Science
