# Supplementary material for: Scaffold-Based Biomaterials for Periodontal Regeneration in Periodontitis: A Systematic Review and Meta-Analysis
Source: J Funct Biomater. 2026 Jun 8;17(6):286. doi: 10.3390/jfb17060286 (PMC13301567; doi:10.3390/jfb17060286)
Supplement: Supplementary file 1 [file jfb-17-00286-s001.zip › Table S1 Literature Search Syntax.pdf]

Supplementary Table S1. Literature Search Syntax

Line #	Searches	Results
MEDLINE (Ovid)		
1	Periodontitis/	24,426
2	periodontitis.ti,ab,kw.	42,903
3	(intrabony or infrabony or "intra bony" or "infra bony" or furcation* or "periodontal defect*").ti,ab,kw.	5,321
4	((vertical or horizontal or "2-wall*" or "3-wall*" or "Class II" or "Class III" or "Class IV") adj3 (defect* or infrabony or furcation*)).ti,ab,kw.	1,957
5	1 or 2 or 3 or 4	54,123
6	Periodontium/	8,642
7	"Guided Tissue Regeneration, Periodontal"/	3,734
8	Alveolar Bone Loss/	12,668
9	6 or 7 or 8	22,941
10	5 and 9	7,069
11	(peri-implant* or periimplant* or implant* or edentulous).ti,ab,kw.	554,262
12	10 not 11	6,014
13	Tissue Engineering/	51,170
14	"Tissue Scaffolds"/	35,116
15	Biodegradable Materials/	-
16	Guided Tissue Regeneration/	2,987
17	Collagen/ or Chitosan/ or Hyaluronic Acid/ or Polyesters/ or Polylactic Acid Polymers/	194,945
18	(scaffold* or matrix or matrices or biomaterial* or hydrogel* or "3D print*" or "3D-printed" or "biofabrication" or "tissue engineer*").ti,ab,kw.	886,953
19	(collagen or chitosan or "hyaluronic acid" or HA or PLA or PLGA or PCL or polycaprolactone or "poly lactic*" or "poly glycolic*" or "beta-tricalcium phosphate" or "beta TCP" or "calcium phosphate*" or alloplast* or xenograf* or allograf*).ti,ab,kw.	682,039
20	("platelet rich*" or PRF or PRP or "growth factor*" or PDGF or BMP-2 or "enamel matrix*" or Emdogain or "small molecule*").ti,ab,kw.	615,776
21	(mesenchymal or MSC or PDL or "periodontal ligament*" or stem cell*).ti,ab,kw.	508,079
22	or/13-21	2,359,741
23	("open flap*" or OFD or debridement or "connective tissue graft*" or FGG or CTG or "acellular dermal*" or ADM or xenogenic).ti,ab,kw.	55,259
24	22 not 23	2,350,362
25	("probing depth*" or PD or PPD or "clinical attachment*" or CAL or VCAL or HCAL or "bone fill*" or "defect fill*" or "radiographic bone*" or "new attachment*" or "new cementum" or "periodontal ligament*" or "new bone*").ti,ab,kw.	296,622
26	Randomized Controlled Trials as Topic/	194,047
27	Random Allocation/	109,409
28	Double-Blind Method/	188,263
29	Single-Blind Method/	36,223
30	Clinical Trial/	538,294
31	(randomized or randomised or randomly or trial or "clinical trial*" or "controlled trial*" or "clinical study*" or "controlled study*").ti,ab,kw.	2,080,629
32	or/26-31	2,515,249
33	12 and 24 and 25 and 32	404
34	limit 33 to (yr="2020 -Current" and english)	92
Embase		
#1	'periodontitis'/exp	67,707
#2	periodontitis:ti,ab,kw	48,294
#3	intrabony OR infrabony OR furcation* OR periodontal defect*	5,693
#4	(vertical OR horizontal OR 2-wall* OR 3-wall* OR class II/III/IV) NEAR/3 (defect* OR infrabony OR furcation*)	2,270
#5	#1 OR #2 OR #3 OR #4	83,657
#6	'periodontium'/exp	59,038
#7	'guided tissue regeneration'/exp	1,213
#8	'alveolar bone loss'/exp	8,186
#9	#6 OR #7 OR #8	65,800

Line #	Searches	Results
#10	#5 AND #9	17,343
#11	peri-implant* OR implant* OR edentulous	789,469
#12	#10 NOT #11	15,520
#13	'tissue engineering'/exp	80,066
#14	biomaterial*	224,403
#15	'tissue scaffold'/exp	26,405
#16	'degradable plastic'	86
#17	'collagen implant'/exp	1,512
#18	'hyaluronic acid'/exp	67,699
#19	'polylactic acid'/exp	16,680
#20	scaffold* OR matrix OR biomaterial* OR hydrogel* OR 3D print* OR tissue engineer*	1,048,672
#21	collagen OR chitosan OR HA OR PLA OR PLGA OR PCL OR beta-TCP OR calcium phosphate OR alloplast* OR xenograft* OR allograft*	901,645
#22	platelet rich* OR PRF OR PRP OR growth factor* OR PDGF OR BMP-2 OR enamel matrix* OR small molecule*	813,121
#23	mesenchymal OR MSC OR periodontal ligament* OR stem cell*	769,343
#24	#13 OR #14 OR #15 OR #16 OR #17 OR #18 OR #19 OR #20 OR #21 OR #22 OR #23	3,144,560
#25	open flap* OR OFD OR debridement OR connective tissue graft* OR FGG OR CTG OR ADM OR xenogenic	74,724
#26	#24 NOT #25	3,131,261
#27	probing depth OR PPD OR CAL OR bone fill OR radiographic bone OR new attachment OR periodontal ligament OR new bone	491,086
#28	'randomized controlled trial'/exp	1,153,999
#29	'controlled clinical trial'/exp	1,345,346
#30	random*	2,605,867
#31	'clinical trial*'	963,282
#32	(clin* NEAR/3 trial*)	1,015,659
#33	(single/double/triple blind* OR mask*)	397,277
#34	placebo*	492,662
#35	#28 OR #29 OR #30 OR #31 OR #32 OR #33 OR #34	3,652,585
#36	#12 AND #26 AND #27 AND #35	499
#37	#36 AND (2020–2026)	218
Cochrane Central Register of Controlled Trials (CENTRAL)		
#1	((periodontitis OR intrabony OR infrabony OR furcation* OR periodontal NEXT defect* OR vertical NEAR/3 defect* OR horizontal NEAR/3 defect* OR Class II NEAR/3 defect* OR Class III NEAR/3 defect*)):ti,ab,kw	10,010
#2	((scaffold* OR biomaterial* OR hydrogel* OR tissue NEXT engineer* OR collagen OR chitosan OR PLA OR PLGA OR PCL OR 3D NEXT print* OR platelet NEXT rich* OR PRF OR PRP OR mesenchymal OR stem)):ti,ab,kw	49,787
#3	((probing NEXT depth* OR PD OR CAL OR bone NEXT fill* OR defect NEXT fill* OR clinical NEXT attachment* OR radiographic NEXT bone* OR new NEXT attachment*)):ti,ab,kw	52,140
#4	((randomized OR randomised OR randomly OR trial OR clinical NEXT trial* OR controlled NEXT trial* OR clinical NEXT study* OR controlled NEXT study*)):ti,ab,kw	1,649,887
#5	#1 AND #2 AND #3 AND #4	556
Web of Science (WoS)		
#1	TS=("Alzheimer Disease" OR Alzheimer* OR "early stage Alzheimer*" OR "early-onset Alzheimer*" OR "early stage dementia" OR "Mild Cognitive Impairment" OR MCI OR (mild NEAR/2 (Alzheimer* OR dementia OR AD)))	378,043
#2	TS=(("aerobic exercise*" OR aerobic* OR cardio* OR "endurance exercise*" OR "endurance training" OR "cardiovascular exercise*") OR ("resistance training" OR "resistance exercise*" OR "strength training" OR "strength exercise*" OR "progressive resistance" OR weightlift* OR "weight training" OR "weight lift*" OR weightbear*))	1,579,779
#3	TS=(cognit* OR "cognitive function*" OR "cognitive performance" OR "cognitive decline" OR "cognitive impairment" OR memory OR "executive function*" OR "executive dysfunction" OR attention OR MMSE OR "mini-mental" OR "mini mental state" OR "ADAS-Cog" OR "neuropsych*" OR "mental processes" OR "neurocognitive")	3,642,922
#4	#1 AND #2 AND #3	8,246
#5	TS=(randomi?ed OR randomized OR "randomised" OR "clinical trial*" OR RCT OR RCTs OR "controlled trial*" OR "randomi?ed controlled trial*" OR "RCT*" OR placebo* OR "double-blind" OR "single-blind" OR "random allocation")	1,971,954
#6	#4 AND #5	1,659
#7	(2020–2026)	804
#8	Document Types: Article	458
